# Cardiovascular outcomes associated with Ultrathin bioresorbable polymer sirolimus eluting stents versus thin, durable polymer everolimus eluting stents following percutaneous coronary intervention in patients with type 2 diabetes mellitus: A meta-analysis of published studies

**DOI:** 10.1097/MD.0000000000023810

**Published:** 2020-12-24

**Authors:** Shibing Deng, Xuying Yi, Zhiming Tian

**Affiliations:** a Department of Internal Medicine, The First Clinical Medical College of Yangtze University; b Department of Internal Medicine, The First People's Hospital of Jingzhou, Jingzhou, Hubei, China.

**Keywords:** Cardiovascular outcomes, Durable polymer everolimus eluting stents, Percutaneous coronary intervention, Stent thrombosis, Type 2 diabetes mellitus, Ultrathin bioresorbable polymer sirolimus eluting stents

## Abstract

**Background:**

Percutaneous coronary intervention with the new generation drug eluting stents (DES) is 1 among the revascularization procedures required to treat patients with coronary artery disease (CAD). Since late stent thrombosis and silent myocardial infarction are highly associated with type 2 diabetes mellitus (T2DM), an analysis comparing the newer generation DES in this specific subgroup of patients would be scientifically relevant.

In this analysis, we aimed to systematically compare the cardiovascular outcomes observed with the ultrathin bioresorbable polymer sirolimus eluting stents (SES) versus thin, durable polymer everolimus eluting stents (EES) following percutaneous coronary intervention in patients with T2DM.

**Methods:**

Through online databases, relevant studies comparing ultrathin bioresorbable polymer SES versus the durable polymer EES were carefully searched. The cardiovascular outcomes were assessed during a follow-up time period of 1 year and more than 1 year (1–5 years) respectively. This meta-analysis was carried out by the latest version of the RevMan software. Following analysis, the results were represented by odds ratios (OR) with 95% confidence intervals (CI).

**Results:**

A total number of 1967 patients with T2DM were included in this analysis. During a 1 year follow-up time period, target lesion failure (TLF) (OR: 0.59, 95% CI: 0.34–1.02; *P* = .06, target vessel revascularization (TVR) (OR: 0.97, 95% CI: 0.55–1.70; *P* = .91) and target lesion revascularization (TLR) (OR: 0.91, 95% CI: 0.44–1.87; *P* = .79) were similarly observed with ultrathin bioresorbable polymer SES versus the thin, durable polymer EES in these patients with T2DM. Other cardiovascular outcomes including myocardial infarction (MI), major adverse cardiac events, all-cause mortality (OR: 0.72, 95% CI: 0.37–1.40; *P* = .34), cardiac death and stent thrombosis (OR: 0.85, 95% CI: 0.45–1.62; *P* = .63) were also similarly observed with these 2 types of new stents. During a follow-up time period above 1 year (1–5 years), still no significant difference was observed in TLF, TVR, TLR, major adverse cardiac events, MI, all-cause mortality, cardiac death and stent thrombosis (OR: 0.62, 95% CI: 0.33–1.16; *P* = .14).

**Conclusions:**

The ultrathin bioresorbable polymer SES were similar to the durable polymer EES in these patients with T2DM. These 2 types of new generation stents were comparable in terms of cardiovascular outcomes. Hence, they might be recommended in patients with T2DM. Upcoming trials should be able to confirm this hypothesis.

## 1 Introduction

Percutaneous coronary intervention (PCI) with the new generation drug eluting stents (DES) is 1 among the revascularization procedures required to treat patients with coronary artery disease (CAD).^[1]^ Previously, 1 major disadvantage of the early generation DES was the delayed healing response of the stented coronary artery which would often result in the occurrence of late stent thrombosis.^[2]^ More precisely, newer DES have thinner struts which have a greater degree of re-endothelization compared to the early DES with thicker struts thereby showing that newer generation DES might be more effective and safe.^[3]^

The new ultrathin biodegradable polymer sirolimus eluting stent (SES) has been compared with the thin, durable polymer everolimus eluting stent (EES).^[4]^ However, these new generation stents have seldom been studied in patients with type 2 diabetes mellitus (T2DM). In addition, previous studies were restricted to a follow-up time period of only 1 year.^[4]^ Observation of outcomes for a longer follow-up duration was rarely studied in patients with T2DM who were revascularized with the new generation stents.

Several recent epidemiological reports have demonstrated a worldwide annual increase in the total number of patients with T2DM. T2DM is associated with high platelet reactivity and inflammatory processes resulting in life threatening consequences.^[5]^ Since diabetes mellitus is among the major risk factors contributing to the high chance for the occurrence of late stent thrombosis and silent myocardial infarction,^[6]^ an analysis comparing the newer generation DES would be scientifically relevant.

In this analysis, we aimed to systematically compare the cardiovascular outcomes observed with the ultrathin bioresorbable polymer SES versus the thin, durable polymer EES following PCI in patients with T2DM at 1 and above 1 year respectively.

## 2 Methods

### 2.1 Data sources

Web of Science, MEDLINE, http://www.ClinicalTrials.com, Cochrane Central, EMBASE and Google Scholar were searched for relevant studies comparing ultrathin bioresorbable polymer SES versus durable polymer EES. The authors also went through references to search for suitable publications.

### 2.2 Search strategies

The following searched terms were used:

(1) Ultrathin bioresorbable polymer SES versus durable polymer EES;
(2) SES versus EES;
(3) Ultrathin bioresorbable polymer SES, durable polymer EES and diabetes mellitus;
(4) New generation drug eluting stents.

Only English publications were searched.

### 2.3 Inclusion and exclusion criteria

Inclusion criteria were:

(1) Studies that compared ultrathin bioresorbable polymer SES versus durable polymer EES;
(2) Studies that consisted of patients with T2DM;
(3) Studies that reported cardiovascular outcomes.

Exclusion criteria were:

(1) Studies that were literature reviews, meta-analyses, systematic reviews or case studies and letters of correspondence;
(2) Studies that did not report cardiovascular outcomes;
(3) Studies that did not involve patients with T2DM;
(4) Studies that did not compare ultrathin bioresorbable polymer SES versus durable polymer EES;
(5) Studies that were associated with the same clinical trial.

### 2.4 Outcomes and definitions

When comparing the cardiovascular events observed between the ultrathin bioresorbable polymer SES versus the thin, durable polymer EES, the following outcomes were assessed:

(1) Target lesion failure (TLF) defined as the composite of clinically driven target lesion revascularization, myocardial infarction or cardiac death related to the target vessel;
(2) Target vessel revascularization (TVR) defined as re-infarction leading to restenting in the previously treated target vessel;
(3) Target lesion revascularization (TLR) which indicates a revascularization procedure with repeated stenting or other revascularization procedures for restenosed, or occluded culprit target lesion;
(4) Myocardial infarction (MI);
(5) Major adverse cardiac events (MACEs) which was defined as the combination of death, MI and revascularization;
(6) All-cause mortality;
(7) Cardiac death;
(8) Stent thrombosis (ST) consisting of definite and probable stent thrombosis.

To have a better analysis, and to avoid mixing all the data irrespective of their follow-up time periods, these cardiovascular outcomes were assessed for a follow-up time period of:

(1) 1 year;
(2) More than 1 year (1–5 years).

Details concerning the outcomes reported in each study and their follow-up time periods have been listed in Table 1.

**Table 1:** Outcomes reported.

Studies	Participants	Outcomes reported	Follow-up time period (yr)
Kandzari 2017^[4]^	T2DM post PCI	TLF, cardiac death, TLR, all-cause mortality, MI, MACEs, ST, Definite ST, Probable ST	1 yr
Kandzari 2018^[7]^	T2DM post PCI	TLF, cardiac death, TLR, all-cause mortality, MI, MACEs, ST, Definite ST, Probable ST, acute ST, sub-acute ST, late ST, very late ST	2 yr
Lansky 2016^[8]^	T2DM post PCI	TLF, TLR, definite and probable ST, cardiac death	1 and 3 yr
Lefevre 2018^[9]^	T2DM post PCI	Death, cardiac death, MI, TLR, TVR, ST, definite ST, probable ST	5 yr
Pilgrim 2018^[10]^	T2DM post PCI	TLF, cardiac death, TLR, all-cause mortality, MI, TVR, MACEs, stroke, definite and probable ST, BARC bleeding type 3–5	5 yr
Roguin 2018^[11]^	T2DM post PCI	TLF, cardiac death, TLR, all-cause death, MI, MACEs, TVR, stent thrombosis, definite and probable ST	1 yr
Yamaji 2018^[12]^	T2DM post PCI	MACEs, all-cause mortality, cardiac death, MI, TLR, TVR, stroke, definite and probable ST	1 yr

### 2.5 Data extraction and quality assessment

Three authors were involved in the data extraction process. The authors’ names, publication year, the corresponding cardiovascular outcomes, the follow-up time periods, the number of patients with T2DM assigned to the SES group and the EES group respectively, the baseline features of the participants, the time period of participants’ enrollment, the methodological features, the types of study, and the antiplatelet drugs which were used were all carefully extracted.

Any disagreement during the data extraction and collection process was further discussed and then a decision was made by the corresponding author.

The quality assessment was carried out by specific tools:

The Cochrane collaboration tool^[13]^ was used to assess the randomized trials whereas the Newcastle Ottawa Scale (NOS)^[14]^ was used to assess the methodological quality of the observational cohorts. Following assessment, a grade was given ranging from grade A implying low risk of bias and grade C implying high risk of bias. Moderate risk was represented by grade B.

### 2.6 Statistical analysis

This meta-analysis was carried out by the latest version of RevMan (Version 5.4). Following analysis, the results were represented by odds ratios (OR) with 95% confidence intervals (CI).

Since heterogeneity is common in meta-analyses, it was assessed by 2 methods:

(1) The *Q* statistic test whereby a subgroup analysis with a *P* value less or equal to .05 was considered statistically significant;
(2) The *I*^2^ statistic test whereby heterogeneity increased with an increasing *I*^2^ percentage.
(3) A fixed effect statistical model (*I*^2^ < 50%) or a random effect statistical model (*I*^2^ > 50%) was used based on the heterogeneity value which was obtained.
(4) Sensitivity analysis was carried out by an exclusion method.

Publication bias was visually observed through funnel plots.

### 2.7 Ethical approval

Ethical approval was not required for this study since data were obtained from previously published studies. The authors were not involved in any experiment carried out on humans or animals for this study.

## 3 Results

### 3.1 Search outcomes

The PRISMA guideline was followed.^[15]^ Since only a few studies were published based on the comparison of ultrathin bioresorbable polymer SES versus thin, durable polymer EES, a total number of only 102 publications were obtained following the search process.

The authors carefully assessed the titles and abstracts. 68 articles were not related to the scope of this study and were therefore eliminated after an initial assessment. 34 full text articles were assessed for eligibility.

Further eliminations of the full text articles were due to the following reasons:

(1) Systematic reviews and meta-analyses/network meta-analyses (2);
(2) Pooled analyses of randomized trials (2);
(3) Case studies (3);
(4) DES were included without specifying which stents were used (EES or another) (2);
(5) Studies involving data which could not be used (2);
(6) Studies based on similar trials (18).

**Figure 1:**
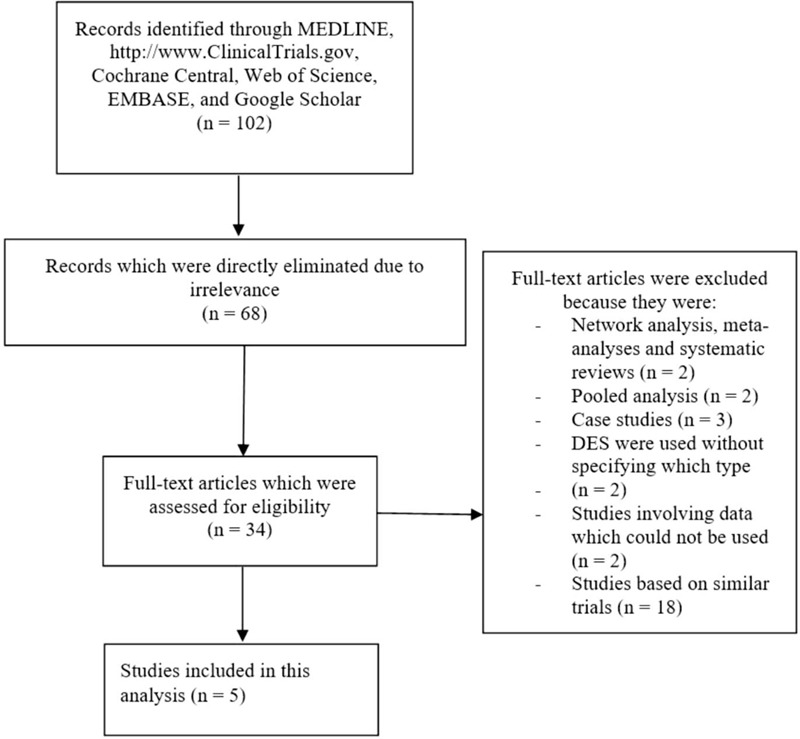
Flow diagram showing the selection of studies.

Finally, only 5 studies^[4,8,9,10,12]^ were included in this meta-analysis (Fig. 1).

### 3.2 Main features of the studies

A total number of 1967 patients with T2DM were included in this analysis. One thousand and 3 (1003) participants were assigned to SES and 964 patients were assigned to EES. Four studies were randomized controlled trials whereas 1 study was an observational cohort. The time period for participant enrollment was between the years 2007 to 2016. The main features of the studies which were included in this analysis have been listed in Table 2.

**Table 2:** Main features of the studies.

Studies	Patients’ enrollment	Type of study	No of T2DM patients with SES (n)	No of T2DM patients with EES (n)	Bias risk grade
Kandzari 2017	2015–2016	RCT	300	166	A
Kandzari 2018^∗^	2015–2016	RCT	300	166	A
Lansky 2016	2007–2013	RCT	30	184	A
Lefevre 2018	2011–2012	RCT	88	44	A
Pilgrim 2018	2012 –2013	RCT	257	229	A
Roguin 2018^∗^	2015 –2016	RCT	154	82	A
Yamaji 2018	2011–2015	OS	328	341	B
Total No of patients (n)			1003	964	

Based on the quality assessment, either a grade A or B was allotted to the studies denoting low and moderate risk of bias respectively.

### 3.3 Baseline features of the participants

Table 3 lists the baseline features of the participants. Among these patients with T2DM, 7.70% to 34.1% were on insulin therapy. The mean age varied from 62.7 to 67.7 years. 70.0 to 78.2% of the participants were males. The percentages of participants with hypertension, dyslipidemia and current smokers were also shown in Table 3.

**Table 3:** Baseline features of the participants.

Studies	Mean age (yr)	Males (%)	HBP (%)	DYS (%)	CS (%)	Insulin requirement (%)
	SES/EES	SES/EES	SES/EES	SES/EES	SES/EES	SES/EES
Kandzari2017	64.5/64.6	75.0/73.0	80.0/80.0	79.0/82.0	24.0/23.0	10.0/11.0
Kandzari2018	64.5/64.6	75.0/73.0	80.0/80.0	79.0/82.0	24.0/23.0	10.0/11.0
Lansky2016	64.5/66.7	70.0/78.0	72.0/68.0	76.0/65.0	20.0/16.0	2.00/9.00
Lefevre2018	62.7/64.8	78.2/74.7	77.5/77.3	67.8/73.4	-	21.4/34.1
Pilgrim2018						
Roguin2018	63.1/63.2	74.2/70.4	76.7/79.8	76.1/82.5	29.5/26.0	10.4/12.6
Yamaji2018	67.7/67.6	74.2/73.3	70.9/71.0	67.6/66.9	27.4/27.5	8.60/7.70

The anti-platelet agents which were used by the participants have been listed in Table 4. In most of the studies, dual antiplatelet therapy with aspirin and clopidogrel was used.

**Table 4:** Antiplatelet agents used.

Antiplatelets used post PCI	Kandzari2017	Kandzari2018	Lansky2016	Lefevre2018	Pilgrim2018	Roguin2018	Yamaji2018
ASA	√	√	√	√	√	√	√
Clopidogrel	√ or	√ or	√	√	√ or	√	√ or
Ticagrelor	√ or	√ or			√ or		√ or
Prasugrel	√	√			√		√

### 3.4 Main analysis

**Figure 2:**
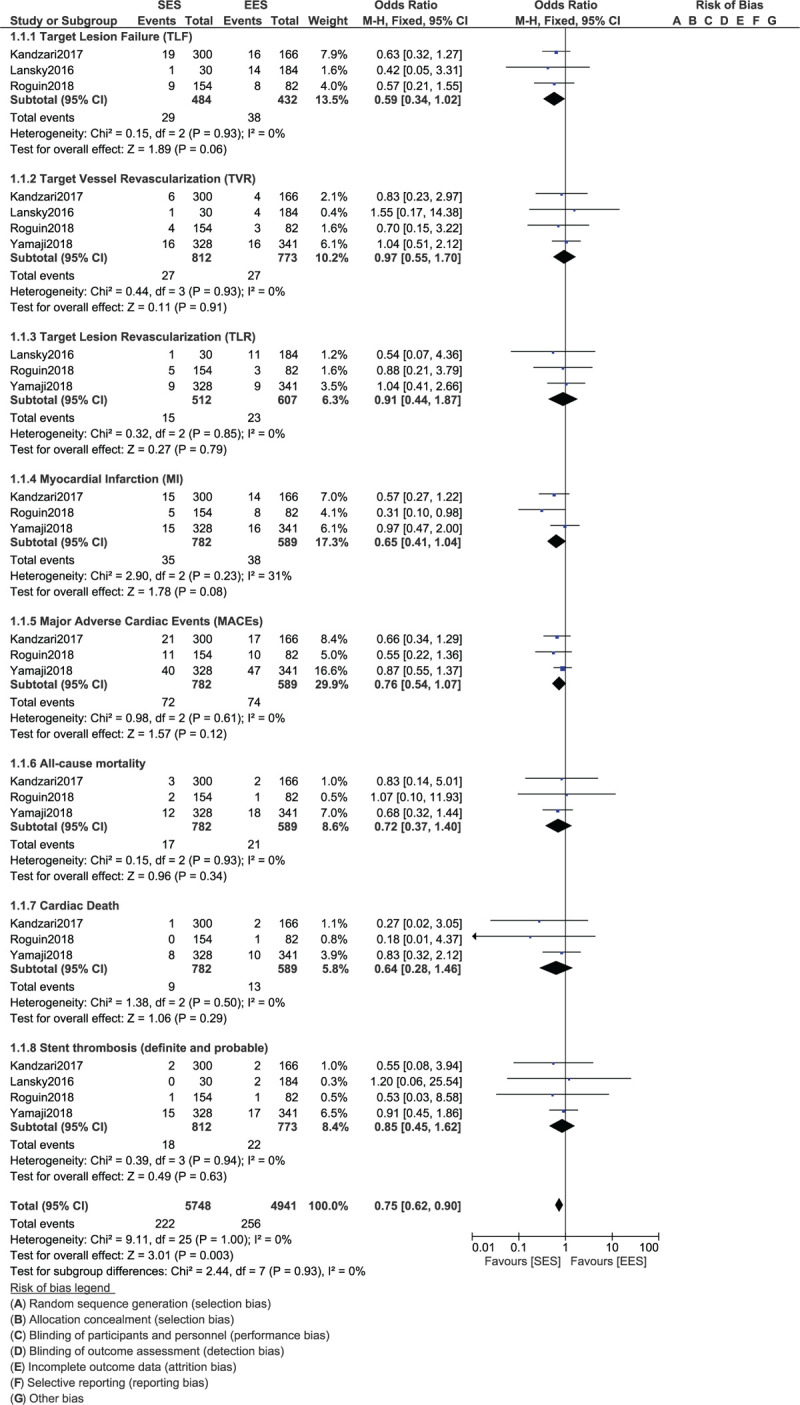
Forest plot showing the comparison of cardiovascular outcomes between ultrathin bioresorbable polymer SES versus durable polymer EES at 1 year.

During a 1 year follow-up time period, TLF (OR: 0.59, 95% CI: 0.34–1.02; *P* = .06, TVR (OR: 0.97, 95% CI: 0.55–1.70; *P* = .91) and TLR (OR: 0.91, 95% CI: 0.44–1.87; *P* = .79) were similarly observed with the ultrathin bioresorbable polymer SES versus the thin, durable polymer EES in these patients with T2DM as shown in Figure 2.

Other cardiovascular outcomes including MI (OR: 0.65, 95% CI: 0.41–1.04; *P* = .08), MACEs (OR: 0.76, 95% CI: 0.54–1.07; *P* = .12), all-cause mortality (OR: 0.72, 95% CI: 0.37–1.40; *P* = .34), cardiac death (OR: 0.64, 95% CI: 0.28–1.46; *P* = .29) and stent thrombosis (OR: 0.85, 95% CI: 0.45–1.62; *P* = .63) were also similarly observed with these 2 types of new stents as shown in Figure 2.

**Figure 3:**
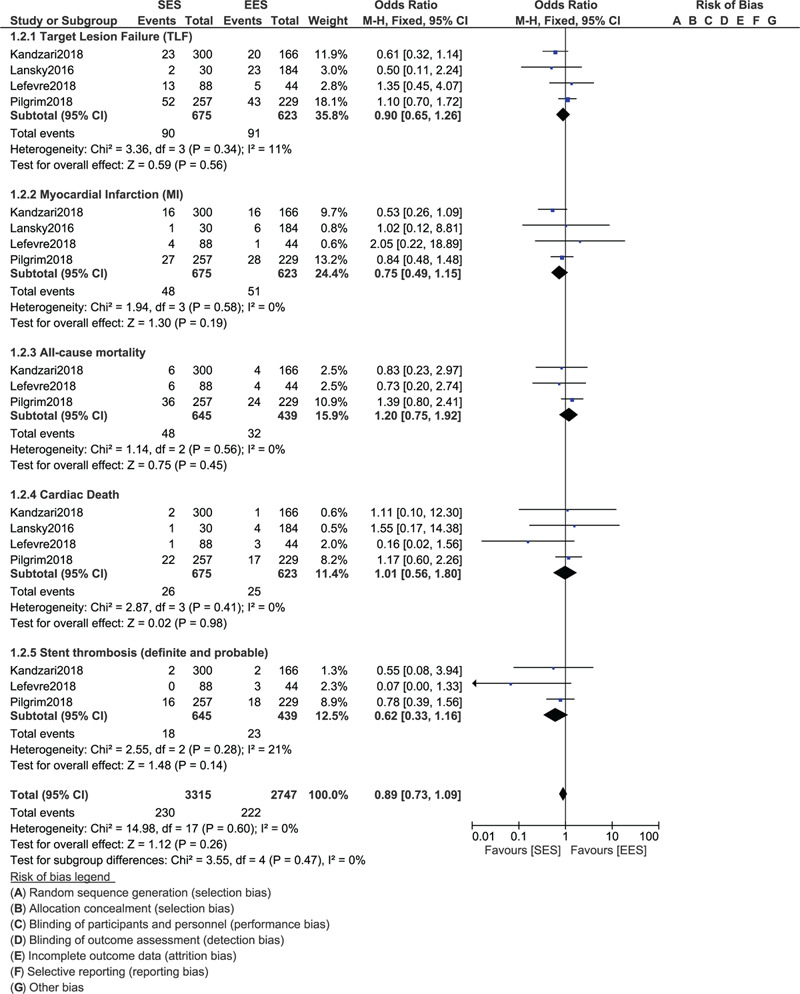
Forest plot showing the comparison of cardiovascular outcomes between ultrathin bioresorbable polymer SES versus durable polymer EES at more than 1 year (part I).

**Figure 4:**
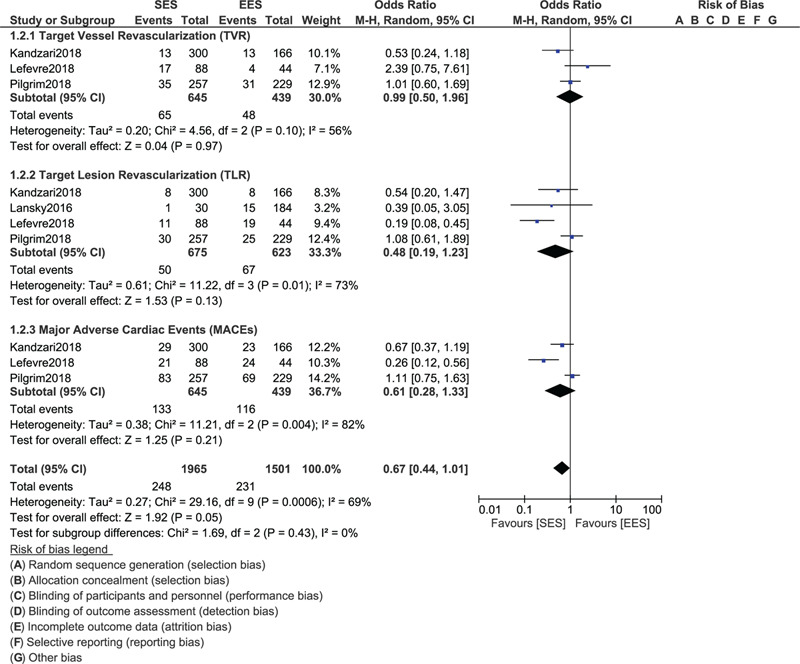
Forest plot showing the comparison of cardiovascular outcomes between ultrathin bioresorbable polymer SES versus durable polymer EES at more than 1 year (part II).

During a follow-up time period above 1 year (1–5 years), still no significant difference was observed in TLF (OR: 0.90, 95% CI: 0.65–1.26; *P* = .56), TVR (OR: 0.99, 95% CI: 0.50–1.96; *P* = .97), TLR (OR: 0.48, 95% CI: 0.19–1.23; *P* = .13), MACEs (OR: 0.61, 95% CI: 0.28–1.33; *P* = .21), MI (OR: 0.75, 95% CI: 0.49–1.15; *P* = .19), all-cause mortality (OR: 1.20, 95% CI: 0.75–1.92; *P* = .45), cardiac death (OR: 1.01, 95% CI: 0.56–1.80; *P* = .98) and stent thrombosis (OR: 0.62, 95% CI: 0.33–1.16; *P* = .14) as shown in Figures 3 and 4.

The results have been summarized in Table 5.

**Table 5:** Results of this analysis.

Outcomes assessed	OR with 95% CI	*P* value	*I*^2^ value (%)
*1 year follow up*			
Target lesion failure	0.59 [0.34–1.02]	.06	0
Target vessel revascularization	0.97 [0.55–1.70]	.91	0
Target lesion revascularization	0.91 [0.44–1.87]	.79	0
Myocardial infarction	0.65 [0.41–1.04]	.08	31
Major adverse cardiac events	0.76 [0.54–1.07]	.12	0
All-cause mortality	0.72 [0.37–1.40]	.34	0
Cardiac death	0.64 [0.28–1.46]	.29	0
Stent thrombosis	0.85 [0.45–1.62]	.63	0
1 yr follow up			
Target lesion failure	0.90 [0.65–1.26]	.56	11
Target vessel revascularization	0.99 [0.50–1.96]	.97	56
Target lesion revascularization	0.48 [0.19–1.23]	.13	73
Myocardial infarction	0.75 [0.49–1.15]	.19	0
Major adverse cardiac events	0.61 [0.28–1.33]	.21	82
All-cause mortality	1.20 [0.75–1.92]	.45	0
Cardiac death	1.01 [0.56–1.80]	.98	0
Stent thrombosis	0.62 [0.33–1.16]	.14	21

### 3.5 Sensitivity analysis and publication bias

**Figure 5:**
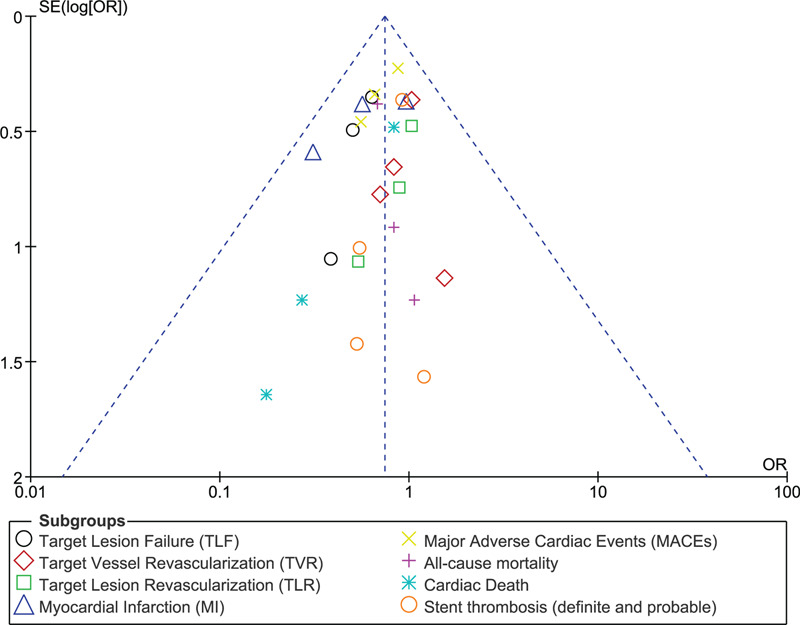
Funnel plot representing publication bias (part A).

**Figure 6:**
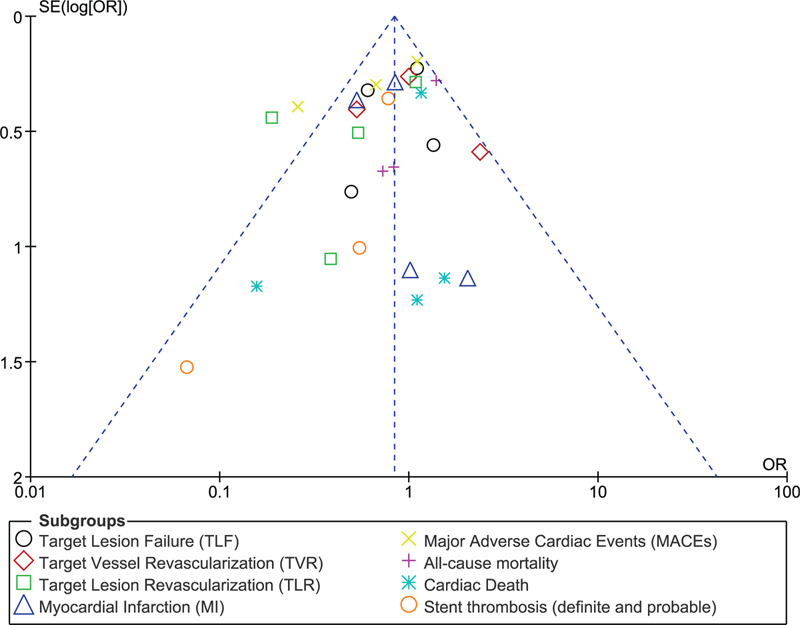
Funnel plot representing publication bias (part B).

In this current analysis, taking into consideration an exclusion method, each study was excluded 1 by 1 and a new analysis was carried out each time. However, consistent results were obtained throughout. Minimal evidence of publication bias was observed through funnel plots as shown in Figures 5 and 6.

## 4 Discussion

In patients with T2DM, our current results showed that the outcomes associated with the ultrathin bioresorbable polymer SES were similar to those of the durable polymer EES following PCI. A similar rate of mortality, target lesion failure, revascularization, MACEs and stent thrombosis was observed with these 2 types of new generation DES.

Similarly, in a recent systematic review and meta-analysis^[16]^ comparing the ultrathin bioresorbable polymer SES versus the thin durable polymer EES, no significant difference was observed in TLF, TVR, cardiac death, all-cause mortality and stent thrombosis. However, the authors concluded that the ultrathin bioresorbable polymer SES was associated with a significantly lower risk of MI when compared to the thin durable polymer EES. However, it should be noted that our analysis included only patients with T2DM whereas the other meta-analysis was based on the general population undergoing PCI.

In another pooled analysis^[17]^ involving only participants with T2DM from the BIOFLOW II, IV and V randomized trials, the authors demonstrated that these 2 new generation DES were associated with similar outcomes further supporting the results of this current analysis. It should also be noted that in their study, the proportion of participants with T2DM on insulin therapy or without insulin was similar.

Our analysis was further supported by the BIOSCIENCE Trial^[18]^ whereby the cardiovascular outcomes which were observed with ultrathin bioresorbable polymer SES were comparable to those from the thin, durable polymer EES during a 2 year follow-up time period. In the BIOSCIENCE Trial, the authors demonstrated that at 2 years, TLF occurred in 10.5% of the participants who were assigned to SES, and in 10.4% of the participants who were assigned to EES.

This current analysis involved only patients with T2DM. Outcomes were assessed during a follow-up time period of 1 year and over 1 year (> 1 to 5 years). There was no significant difference in outcomes between SES and EES throughout this analysis. However, future trials with larger number of participants with T2DM will have to confirm this hypothesis.

### 4.1 Limitations

This current analysis has limitations. First of all, not many research papers were published comparing the ultrathin bioresorbable polymer SES versus the thin, durable polymer EES following PCI in patients with T2DM. Hence, the total number of participants were limited for this analysis. Important outcomes such as acute, sub-acute and late stent thrombosis could not be assessed since these endpoints were reported in only 1 original study. Even though each original study mentioned the antiplatelet agents which had been used, bleeding outcomes were reported only in 1 study and therefore, a subgroup analysis of the bleeding events was not possible. Another limitation of this study was the fact that duration of antiplatelet therapy was not available.

## 5 Conclusions

The ultrathin bioresorbable polymer SES were similar to the durable polymer EES in these patients with T2DM. These 2 types of new generation stents were comparable in terms of cardiovascular outcomes. Hence, they might be recommended in patients with T2DM. Upcoming trials should be able to confirm this hypothesis.

## Author contributions

The authors Dr Shibing Deng, Dr Xuying Yi and Dr Zhiming Tian were responsible for the conception and design, acquisition of data, analysis and interpretation of data, drafting the initial manuscript and revising it critically for important intellectual content. Dr Shibing Deng and Dr Xuying Yi are the first co-authors and they wrote this manuscript. All the authors agreed and approved the manuscript as it is.

**Conceptualization:** Shibing Deng, Xuying Yi, Zhiming Tian.

**Data curation:** Shibing Deng, Xuying Yi, Zhiming Tian.

**Formal analysis:** Shibing Deng, Xuying Yi, Zhiming Tian.

**Funding acquisition:** Shibing Deng, Xuying Yi, Zhiming Tian.

**Investigation:** Shibing Deng, Xuying Yi, Zhiming Tian.

**Methodology:** Shibing Deng, Xuying Yi, Zhiming Tian.

**Project administration:** Shibing Deng, Xuying Yi, Zhiming Tian.

**Resources:** Shibing Deng, Xuying Yi, Zhiming Tian.

**Software:** Shibing Deng, Xuying Yi, Zhiming Tian.

**Supervision:** Shibing Deng, Xuying Yi, Zhiming Tian.

**Validation:** Shibing Deng, Xuying Yi.

**Visualization:** Shibing Deng, Xuying Yi, Zhiming Tian.

**Writing – original draft:** Shibing Deng, Xuying Yi.

**Writing – review & editing:** Shibing Deng, Xuying Yi.
